# Editorial: Recent advances in carbohydrate chemical and enzymatic syntheses

**DOI:** 10.3389/fchem.2023.1144042

**Published:** 2023-01-25

**Authors:** M. Carmen Galan, Suvarn S. Kulkarni, Clay S. Bennett

**Affiliations:** ^1^ School of Chemistry, University of Bristol, Bristol, United Kingdom; ^2^ Department of Chemistry, Indian Institute of Technology Bombay, Mumbai, India; ^3^ Department of Chemistry, Tufts University, Medford, MA, United States

**Keywords:** carbohydrates, glycoconjugates, chemoenzymatic, synthesis, oligosaccharides, glycopeptides, natural products

It is now widely accepted that oligosaccharides and glycoconjugates play a host of critical roles in biological processes, including cell adhesion, signaling, and the immune response (Varki, 1993). This has led to increased recognition that glycoconjugates with unusual glycosylation patterns hold enormous potential for developing vaccines against a variety of diseases ranging from pathogens to cancer (Astronomo and Burton, 2010; Berti and Adamo, 2018). Even free oligosaccharides have been shown to possess potent bioactivity such as the antimicrobial activity of human milk oligosaccharides (HMOs) (Craft and Townsend, 2019) and the myriad of roles that heparan sulfate plays in cell-cell interactions (Chhabra et al., 2021). Furthermore, the alteration of carbohydrates on natural products can dramatically alter their activity, a process known as glycodiversification (Elshahawi et al., 2014).

Before the true promise of carbohydrates in therapeutic development can be realized, however, it will be necessary to develop robust and reliable methods for their synthesis. This Research Topic presents a series of original research articles and reviews describing methods for the chemical and chemoenzymatic synthesis of mammalian and prokaryotic glycans, glycopeptides and glycosylated natural products. The seven contributions from top research groups in this volume includes six original research articles and one review.

Since carbohydrates are often produced as complex mixtures, synthesis is therefore the only way to produce appreciable amounts of pure material for study. When they can be used, glycosyltransferase enzymes can provide remarkable regio- and stereoselectivity in oligosaccharide synthesis. This is elegantly exemplified in original research from Ooi et al. where they describe a chemoenzymatic approach to lacto-*N*-hexose, a major component of antimicrobial human milk oligosaccharides. In this approach, the authors first use chemical synthesis to obtain the tetrasaccharide core of this molecule. Enzymatic elaboration of this molecule using the galactotransferases HP0826 and WbgO then affords the target structure.

Whilst enzymatic synthesis is incredibly powerful, enzymes are not always readily available, especially for prokaryotic glycosides and non-native structures. As a result, total chemical synthesis remains an important tool for glycoside production. This is demonstrated in original research by Zhang et al. through the synthesis of a library of defined cationic Pel polysaccharides from *Pseudomonas aeruginosa.* Through an elegant linear approach, the authors can produce fragments up to a heptasaccharide, composed entirely of 1,2-*cis*-linkages. In another example of the power of synthesis, Tseng et al. describe the preparation of unnatural heparin trisaccharide fragments possessing modification at the 6-position of the central residue. Through this work, they can obtain several inhibitors of human endo-6-*O*-sulfatase 1, which is overexpressed in cancer cells.

Another challenge in oligosaccharide synthesis lies in the fact that many monosaccharides are not readily available. This is especially true for monosaccharides from prokaryotes and natural products. In an original research article, Novakova et al. describe a synthetic route to the rare sugar D-altrosamine from glucose. The authors also demonstrate it is possible to control selectivity in glycosylation reactions with this compound through the installation of a directing picoloyl ester at the C3 position of this molecule. These unusual sugars can also serve as precursors for higher sugars as exemplified by original research from Zhou et al. here the authors describe a route to 2,3,6-trideoxy-2,6-diacetamido sugars from common monosaccharides. The resulting products can then be transformed into nine carbon non-ulosonic acids using the pseudaminic acid synthetase.

Glycoconjugates, in which a carbohydrate is attached to another molecule such as a peptide or natural product also possess potent biological activity. Dolan et al. describe an approach to functionalized glycopeptides through conjugation of a glycosylated MUC1 peptide to cholera toxin B subunit (CTB) using the enzyme sortase. This approach allows for selective labeling of the C-terminus of a peptide, leaving the N-terminus free for further conjugation. In the context of glycosylated small molecules, Wang et al. provide an excellent review on the work directed toward the synthesis of resin glycosides over the past several years. This manuscript nicely exemplifies the challenges associated with the construction of glycosylated natural products.

We believe that the work described in this Research Topic nicely highlights some of the current challenges and progress that exist in the field of carbohydrate chemistry. It is our hope that this work will encourage other chemists to toss their own hat into this exciting and rapidly growing field, where there are many more discoveries to be made.
